# Genomic insights into low-level rifampicin resistance mediated by borderline *rpoB* mutations in *Mycobacterium tuberculosis*: prevalence and phylogeny in Northeast China

**DOI:** 10.1128/spectrum.00327-26

**Published:** 2026-07-21

**Authors:** Lingling Li, Mingjin Zhang, Lin Su, Xiaoting Qin, Huiying Liu, Xue Zhang, Kewei Liu, Xuejuan Sun, Feng Yan

**Affiliations:** 1 Department of Clinical Laboratory, Changchun Infectious Disease Hospital 117849 , Changchun, China; 2 School of Basic Medical Sciences, Beihua University 74568, Jilin, China; University of Texas Medical Branch at Galveston, Galveston, Texas, USA

**Keywords:** low-level resistance, whole-genome sequencing, molecular epidemiology, diagnostic discordance

## Abstract

The emergence of low-level rifampicin (RIF) resistance in *Mycobacterium tuberculosis* poses a challenge to tuberculosis (TB) control, as it often leads to discordance between genotypic resistance detected by molecular assays (e.g., Xpert MTB/RIF) and phenotypic susceptibility in conventional drug susceptibility testing (DST). In this study, we performed whole-genome sequencing (WGS) on 17 clinical isolates from Changchun, Northeast China, which exhibited such discordance. All isolates harbored functional borderline mutations in the *rpoB* RRDR region, predominantly *Leu452Pro* and *Leu430Pro* (29% each), followed by *His445Asn* (18%). RIF minimum inhibitory concentration (MIC) values ranged from ≤0.25 to 1.0 mg/L, confirming low-level resistance. Notably, 53% (9/17) of the isolates were co-resistant to fluoroquinolones and 24% (4/17) to isoniazid (INH). According to WHO classification, 59% (10/17) were pre-extensively drug-resistant TB (Pre-XDR-TB) or multidrug-resistant TB (MDR-TB). Phylogenetic analysis revealed that 94% (16/17) belonged to the East Asian Beijing lineage (Lineage 2.2.1), with no evidence of recent local transmission. These findings underscore the complexity of low-level RIF resistance and its frequent association with broader drug resistance in a dominant lineage, highlighting the need for integrating MIC and WGS into diagnostic algorithms to guide appropriate treatment and surveillance.

**IMPORTANCE:**

The accurate detection of RIF resistance is critical for the management of TB, yet standard phenotypic methods often fail to identify strains with low-level resistance conferred by borderline *rpoB* mutations. This study provides the first genomic characterization of such discordant isolates in Northeast China, revealing a high prevalence of co-resistance to other key drugs and a strong association with the locally dominant Beijing lineage. The findings emphasize that reliance on phenotypic DST alone may lead to underestimation of drug resistance and inappropriate treatment, potentially contributing to the emergence and spread of Pre-XDR-TB and MDR-TB. Incorporating MIC determination and WGS into routine diagnostics could enhance detection, inform tailored therapy, and improve surveillance of these clinically significant strains.

## INTRODUCTION

TB remains a major global public health threat, and its effective control relies heavily on accurate identification and timely intervention against drug-resistant strains. RIF, a cornerstone of first-line anti-TB treatment, serves as a key marker for diagnosing MDR-TB and directly influences treatment success (1). In clinical practice, the detection of RIF resistance primarily relies on two technical approaches: molecular rapid tests represented by Xpert MTB/RIF, which enable fast genotypic screening by targeting the RRDR of the *rpoB* gene; and phenotype-based drug susceptibility testing (pDST) founded on conventional culture techniques, such as Löwenstein–Jensen (L-J) medium-based DST, which evaluates the inhibitory effect of the drug on bacterial growth through *in vitro* culture. Although results from these methods are generally concordant, an increasing number of cases show molecular resistance but phenotypic susceptibility, creating a pressing challenge for result interpretation and clinical decision-making.

With the widespread use of molecular testing for RIF resistance screening, various rare mutations associated with resistance (AwR) have been identified. Among these, borderline mutations in *rpoB* are of particular interest. These mutations cause only slight increases in RIF MIC, placing them near or just below the clinical breakpoint, thereby conferring low-level or borderline resistance (2). Emerging evidence suggests that such mutations have clinical significance, as they have been associated with unfavorable treatment outcomes when RIF is included in the regimen (3, 4). However, systematic data on the global prevalence of low-level RIF resistance in *Mycobacterium tuberculosis* (*M. tuberculosis*), especially in Northeast China, remain scarce. Changchun, as the capital of Jilin Province and a major city in Northeast China, bears a representative TB burden in the region. Reported prevalence of these mutations varies geographically (5–7). Furthermore, some rare RRDR variants may be missed or misinterpreted in routine testing, potentially leading to underestimation or underreporting.

Notably, the co-resistance patterns of low-level RIF-resistant strains remain controversial. Some studies report that such resistance frequently presents as mono-resistance (8), while others indicate frequent co-occurrence with INH resistance, constituting MDR-TB (9, 10). Accurate identification of resistance patterns is clinically crucial, as it directly affects treatment selection and prognosis. Additionally, the evolution of drug resistance in *M. tuberculosis* is closely linked to phylogenetic lineage. Lineage 2, predominant in China, has been associated with the accumulation of multidrug resistance (11–13). This suggests that the characteristics and transmission potential of low-level RIF resistance may be influenced by lineage background.

Despite recognition of the importance of lineage background, knowledge of strains carrying borderline mutations remains limited. First, the full spectrum of mutations, precise MIC distributions, and mechanisms underlying phenotypic discordance need confirmation in larger clinical cohorts using high-resolution methods. Second, whether such borderline resistance is associated with specific *M. tuberculosis* lineages, particularly high-prevalence lineages like Lineage 2, and its distribution and evolutionary significance remain unclear. Moreover, whether these strains frequently carry resistance to other anti-TB drugs, leading to more complex phenotypes such as Pre-XDR-TB, and whether they form transmission clusters in the community are critical for public health interventions but require systematic investigation using WGS.

Therefore, we hypothesized that in Changchun, Northeast China, *M. tuberculosis* isolates showing genotypic-phenotypic discordance are mainly caused by borderline *rpoB* mutations, often accompanied by other drug resistance, primarily belong to the locally prevalent Lineage 2, and have not formed significant local transmission chains. To test this hypothesis, we conducted a retrospective study with the following objectives: (i) to clarify the spectrum of borderline *rpoB* mutations and their precise MIC distributions, (ii) to reveal the phylogenetic lineage composition and co-resistance patterns of these strains, and (iii) to assess genetic evidence of recent transmission based on core genome SNP analysis. As the first WGS-based systematic characterization of such discordant strains in Northeast China, this study provides key scientific evidence for improving laboratory diagnostics, guiding precision therapy, and informing regional control strategies.

## MATERIALS AND METHODS

### Study isolates

This retrospective study included clinical isolates of *M. tuberculosis* collected in Changchun from 1 January 2024 to 31 October 2025. From an initial cohort of 1,238 isolates, we identified the final study isolates through the following steps. First, all isolates with RIF resistance by Xpert MTB/RIF were selected. These were then subjected to L-J proportion method pDST, and only those with phenotypic susceptibility to RIF were retained. Twenty isolates initially met the discordance criteria.

To ensure reliable genotypic resistance results, all candidate isolates were validated. First, the RIF MIC was determined by broth microdilution (BMD) and confirmed to be ≤1 µg/mL for all isolates. Second, follow-up Xpert MTB/RIF testing using pure cultures yielded negative RIF resistance results in three isolates, which conflicted with the initial test outcomes. Their initial bacillary loads were one “low” and two “very low.” We attribute this discrepancy to cut-off errors. When the bacillary load is close to the limit of detection (LoD) of the assay, unstable amplification and hybridization signals may produce inconsistent positive or negative readings with poor test reproducibility and further give rise to false resistance calls (14, 15). Accordingly, the three isolates were excluded to guarantee the accuracy of genotypic data.

Finally, 17 isolates with confirmed MIC and reliable genotypic resistance results constituted the core analysis cohort for WGS and further analysis.

### Laboratory methods

#### Xpert MTB/RIF testing

The Cepheid Xpert MTB/RIF assay was performed according to the manufacturer’s instructions. Clinical samples (≥1 mL) were treated with sample reagent, mixed thoroughly, and tested.

### Bacterial culture and identification

Mycobacterial culture was performed using the BACTEC MGIT 960 system. Positive cultures were initially identified by Ziehl–Neelsen staining, and *M. tuberculosis* complex was confirmed using pnitrobenzoic acid medium (BASO Diagnostics, Zhuhai, China). The proportion method was performed using drug-containing *Mycobacterium tuberculosis* Löwenstein–Jensen (L-J) medium (Encode Medical, Zhuhai, China). The tested drugs and their corresponding critical concentrations were as follows: RIF (40 mg/L), INH (0.2 mg/L), ethambutol (EMB, 2 mg/L), levofloxacin (LFX, 2 mg/L), moxifloxacin (MFX, 2 mg/L), amikacin (AMK, 30 mg/L), prothionamide (PTO, 40 mg/L), streptomycin (SM, 4 mg/L), and para-aminosalicylic acid (PAS, 1 mg/L). *Mycobacterium tuberculosis* H37Rv was used as the quality control strain in each batch of tests.

### MIC determination

MIC was determined by BMD using commercial plates (BASO Diagnostics, Zhuhai, China). The plates contained RIF at serial concentrations of 0.25, 0.5, 1, 2, 4, and 8 mg/L. Revisions to the critical concentrations of commercial kits require registration verification and clinical trials, and the updated criteria are still pending final approval by domestic regulatory authorities. Thus, we adopted the original breakpoints specified by the kit. Several breakpoints in this study differed from the epidemiological cut-off values recommended by the WHO: RIF (1 mg/L vs 0.5 mg/L), INH (0.2 mg/L vs 0.125 mg/L), EMB (5 mg/L vs 4 mg/L), and LFX (2 mg/L vs 1 mg/L). According to the MIC profiles of the study isolates, five strains harboring the *rpoB Leu452Pro* mutation (MIC = 1 mg/L) would be classified as RIF-resistant if the WHO criteria were applied. No misclassification of susceptibility or resistance was observed for other drugs among all tested isolates. The full data set of L-J drug susceptibility results and BASO-derived MIC values for each drug is presented in Table S1. Detailed information on all tested drugs, corresponding concentrations on assay plates and interpretive breakpoints, is listed in Table S2.

Drug susceptibility was interpreted as susceptible when MIC ≤ critical concentration and resistant when MIC > critical concentration. For PZA, isolates were defined as susceptible (S) if bacterial growth in the detection well was reduced by 50% compared with the PZA positive control well; otherwise, the result was recorded as resistant (R). *M. tuberculosis* H37Rv was included as a quality control strain in all three batches of BMD testing. Its RIF MIC remained consistently below 0.25 mg/L in every run.

### WGS and data analysis

Genomic DNA was extracted using the CTAB method. PCR-free libraries with insert sizes of 300–500 bp were constructed and sequenced on the DNBSEQT7 platform with a paired-end 150 bp (PE150) strategy. *De novo* assembly was performed using SPAdes 3.13.0 (Assembly Analysis). Average sequencing depth was ≥100× , genome coverage ≥ 95%, and Q30 ≥85%. Using *M. tuberculosis* H37Rv as the reference genome, TB-Profiler (https://tbdr.lshtm.ac.uk/) was used for resistance-associated mutation and lineage analysis.

### Phylogenetic and transmission analysis

A maximum-likelihood phylogenetic tree was constructed using FastTree based on core genome SNPs. Isolates with ≤5 core genome SNP differences and located within the same high-support branch were considered a genetic cluster indicative of recent transmission (16).

### Descriptive statistics

Categorical variables (mutation types, resistance rates, demographic characteristics) were described as frequencies (percentages). Given the small sample size, all percentages were rounded to the nearest integer. Continuous variables (e.g., MIC values) were presented as ranges.

## RESULTS

### Characteristics of the study cohort

We retrospectively included 1,238 clinical isolates of *M. tuberculosis* collected between 1 January 2024 and 31 October 2025. The screening process yielded 20 isolates with genotypic resistance but phenotypic susceptibility to RIF. After excluding 3 isolates due to heteroresistance-related false positives, 17 isolates (1.37% of the initial cohort) were included in the final analysis.

All patients were from Changchun. Demographic and clinical characteristics were as follows: 14 (82%) were male and 3 (18%) female; age was mainly 40–59 years (9, 53%), followed by ≥60 years (5, 29%) and 20–39 years (3, 18%). Eight patients (47%) were newly diagnosed, and nine (53%) had a history of previous treatment. Comorbidities included diabetes (3, 18%), chronic obstructive pulmonary disease or other chronic lung diseases (3, 18%), and HIV infection (2, 12%).

### RIF MIC distribution

BMD results showed that RIF MICs for all 17 isolates were ≤1.0 mg/L, ranging from ≤0.25 to 1.0 mg/L (Table 1). No isolate had an MIC above the clinical breakpoint (1 mg/L).

**TABLE 1:** Drug susceptibility profiles and major lineage distribution of *Mycobacterium tuberculosis* isolates harboring *rpoB* resistance mutations^*a*^

Mutation (*rpoB*)	RIF MIC (mg/L)	Other mutations (*n*)	DR-TB subtypes (*n*)	Confidence grade (*rpoB*)	Lineage (*n*)			
					1	2	3	4
*Leu452Pro*	1.0	ND (2)	RR-TB (2)	AwR	2			
	1.0	*gyrA Asp94Ala* (2)	Pre-XDR-TB (2)		2			
	1.0	*katG Ser315Asn; gyrA Asp94Gly* (1)	Pre-XDR-TB (1)		1			
*Leu430Pro*	≤0.25	*embB Met306Ile* (1)	RR-TB (1)	AwR	1			
	0.5	*inhA c.-777C > T*; *gyrA Asp94Ala*; *rpsL Lys43Arg* (1)	Pre-XDR-TB (1)		1			
	0.5	*gyrA Asp94Asn* (1)	Pre-XDR-TB (1)		1			
	0.5	*katG Ser315Thr; pncA Thr47Ala; gyrA Asp94Asn*; *rpsL Lys43Arg* (1)	Pre-XDR-TB (1)		1			
	≤0.25	*gyrA Asp94Gly* (1)	Pre-XDR-TB (1)		1			
*His445Asn*	≤0.25	*rpsL Lys43Arg* (2)	RR-TB (2)	AwR	2			
	≤0.25	*katG Ser315Thr*; *gidB c.102delG* (1)	MDR-TB (1)					1
*Leu452Met*	≤0.25, 0.5	ND (2)	RR-TB (2)	Interim	2			
*Asp435Ala*	≤0.25	*gyrA Asp94Gly* (1)	Pre-XDR-TB (1)	Interim	1			
*Asp435Gly*	≤0.25	*gyrA Asp94Gly* (1)	Pre-XDR-TB (1)	Interim	1			

^*a*^ Lineage: 1, Indo-Oceanic; 2, East Asian (Beijing); 3, East African-Indian; 4, Euro-American. Abbreviations: RIF, rifampicin; DR-TB, drug-resistant tuberculosis; RR-TB, rifampicin-resistant tuberculosis; MDR-TB, multidrug-resistant tuberculosis; Pre-XDR-TB, pre-extensively drug-resistant tuberculosis; AwR, Associated with resistance; Interim, Associated with resistance-Interim; ND, not detected. Numbers in parentheses indicate the number of isolates. Confidence Grade, initial confidence classification of the association between mutation and drug resistance.

### Genotypic characteristics

WGS analysis revealed functional nonsynonymous mutations (referring to amino acid-altering variants with confirmed or potential functional impacts such as drug resistance) (17) in the RRDR of *rpoB* in all 17 isolates. The predominant mutations were *Leu452Pro* and *Leu430Pro* (29% each), followed by *His445Asn* (18%, 3/17), *Leu452Met* (12%, 2/17), *Asp435Ala* (6%, 1/17), and *Asp435Gly* (6%, 1/17).

**Fig 1:**
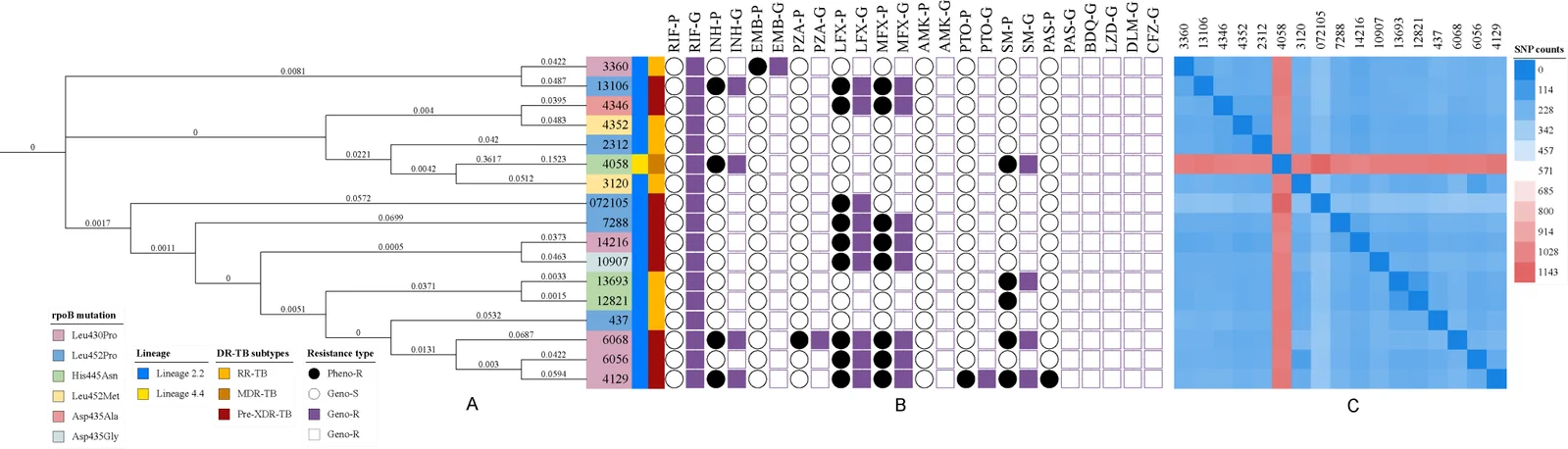
Phylogenetic Tree with Lineage (**A**), Drug Resistance Profiles (**B**), and SNP Distance Heatmap (**C**) of 17 rifampicin-resistant *Mycobacterium tuberculosis* clinical isolates. Abbreviations: RR-TB, rifampicin-resistant tuberculosis; MDR-TB, multidrug-resistant tuberculosis; Pre-XDR-TB, pre-extensively drug-resistant tuberculosis; Pheno, phenotype; Geno, genotype; R, resistant; S, susceptible; RIF, rifampicin; INH, isoniazid; EMB, ethambutol; PZA, pyrazinamide; LFX, levofloxacin; MFX, moxifloxacin; AMK, amikacin; PTO, prothionamide; SM, streptomycin; PAS, para-aminosalicylic acid; BDQ, bedaquiline; LZD, linezolid; DLM, delamanid; CFZ, clofazimine; Pheno, phenotype; Geno, genotype; S, susceptible; R, resistant. Note: Values on tree branches indicate branch lengths corresponding to genetic distances derived from core-genome SNPs.

Regarding co-resistance, 53% (9/17) carried mutations in the quinolone resistance-determining region of *gyrA*, mainly at *Asp94 (Asp94Gly* in 4, *Asp94Ala* in 3, and *Asp94Asn* in 2); no *gyrB* mutations were detected. INH resistance-associated mutations were found in 24% (4/17), including *katG Ser315Thr* (2), *katG Ser315Asn* (1), and the *inhA c.-777C > T* (1). Additionally, 24% (4/17) had streptomycin resistance mutations (*rpsL Lys43Arg* or *gidB*). One isolate each (6%) had *embB Met306Ile* (ethambutol) and *pncA Thr47Ala* (pyrazinamide, PZA) mutations. No mutations associated with resistance to amikacin, bedaquiline, linezolid, delamanid, or clofazimine were detected (Fig. 1).

Phenotypic resistance results were highly consistent with genotypic findings: all fluoroquinolone-resistant isolates carried *gyrA* mutations, and all INH-resistant isolates had *katG* or *inhA* mutations. Notably, one isolate was phenotypically resistant to streptomycin and another to para-aminosalicylic acid, with no relevant resistance genes identified.

### Phenotypic resistance profiles and drug resistance categories

Phenotypic susceptibility to 10 anti-tuberculosis drugs was tested. Nine drugs were examined using the L-J proportion method, while PZA was detected via the BMD method. Among first-line drugs, the resistance rate of INH was 24% (4/17). Ethambutol and PZA each showed a resistance rate of 6% (1/17). Among second-line drugs, fluoroquinolone resistance was prominent, with levofloxacin and moxifloxacin resistance rates of 53% (9/17) and 47% (8/17), respectively. All isolates were phenotypically susceptible to RIF, consistent with the study inclusion criteria.

According to WHO classification (1), combining genotypic (RIF) and phenotypic (other drugs) results, the resistance categories were as follows: RIF-resistant TB (RR-TB), seven isolates (41%); Pre-XDR-TB, nine isolates (53%); MDR-TB, one isolate (6%). Overall, 59% (10/17) were Pre-XDR-TB or MDR-TB.

### Phylogenetic characteristics and transmission analysis

The phylogenetic tree based on core genomes showed that 94% (16/17) belonged to Lineage 2.2.1, and only one isolate belonged to the Euro-American lineage (Lineage 4.4.2) (Fig. 1).

Pairwise core-genome SNP differences were all >5. No cluster with ≤5 SNP differences within a high-support branch was observed, indicating no evidence of recent transmission among the study isolates.

## DISCUSSION

### Prevalence of borderline *rpoB* mutations and limitations of phenotypic testing

Our data evidence that RRDR-located functional nonsynonymous *rpoB* variants are the primary driver of discordant RIF susceptibility results, with *Leu452Pro* and *Leu430Pro* as the dominant causal variants. These findings align with previous studies indicating that these mutations are the main cause of low-level RIF resistance and phenotypic underestimation (8, 18, 19).

Variable RIF MIC distributions across these mutations underpin discrepant testing outcomes. BMD reliably identifies borderline resistance via serial drug gradients, whereas L-J proportion methods are restricted by fixed incubation cycles and uneven drug diffusion; strains with modestly reduced growth often fail to meet solid-medium resistance cutoffs and are incorrectly categorized as susceptible (18), explaining the clinical inconsistency of Xpert-defined resistance but culture-susceptible results.

Molecular mechanisms for such false-susceptible phenotypes fall into two core categories. First, variants including *Leu452Pro* induce partial conformational changes in the *rpoB* target, moderately decreasing rifampin binding affinity to elevate MIC without triggering high-grade resistance (20); molecular simulation data confirm proline substitution at codon 452 destabilizes rifampin-target interaction without fully abolishing drug binding (21). Second, these mutations generally impair *in vitro* bacterial fitness, slowing isolate proliferation and preventing achievement of solid-medium growth thresholds within standard culture durations (18, 22).

WHO has formally established a composite criterion for defining RIF resistance. A strain is identified as RIF-resistant if it exhibits phenotypic resistance, carries any nonsynonymous mutation in the *rpoB* RRDR, or harbors one of the seven clinically relevant borderline *rpoB* mutations. These seven variants include *Leu430Pro*, *Asp435Tyr*, *His445Leu*, *His445Asn*, *His445Ser*, *Leu452Pr*o, and *Ile491F* (23). Three variants detected in our study, namely, *Leu430Pro*, *His445Asn,* and *Leu452Pro*, belong to the above list and are categorized as AwR in the WHO mutation catalog (17). Despite the same AwR classification, these variants display distinct phenotypic profiles against the WHO-recommended liquid-medium breakpoint of 0.5 mg/L (2, 24). Specifically, *Leu452Pro* has an MIC above this threshold and meets the criteria for phenotypic resistance, while *Leu430Pro* and *His445Asn* show borderline MIC values close to the cutoff. The remaining variants *Asp435Ala*, *Asp435Gly,* and *Leu452Met* are classified as Interim, and their clinical significance requires further validation in larger clinical cohorts.

### Complexity of resistance profiles: Composite resistance patterns and association with lineage 2 background

In the present study, isolates with borderline *rpoB* mutations frequently displayed additional drug resistance, and approximately 60% were defined as Pre-XDR/MDR-TB in accordance with WHO criteria. Such frequent combined resistance differs sharply from Lineage 4 isolates described in a US-based investigation, which mostly present isolated RIF monoresistance (8), pointing to lineage- and geography-dependent resistance features. Despite the predominant Lineage 2 membership consistent with reports from southern China, several region-specific *rpoB* substitutions documented in southern isolates were absent in our strains (19). Overall, borderline *rpoB* mutations exhibit clear regional variation even within the Beijing lineage, presumably shaped by local antimicrobial selection pressure and sublineage microevolution.

Lineage 2 predominance is consistent with its high prevalence in China; the overwhelming predominance of Lineage 2 matches its nationwide epidemiological dominance in China (25, 26). Enhanced genetic plasticity of Beijing-family strains facilitates easier accumulation of drug-resistance mutations (27), which helps explain the abundant co-resistance observed herein. Core-genome SNP analysis revealed no clustered transmission events among borderline mutants, in line with the hypothesis that borderline mutations impose fitness costs and constrain bacterial transmissibility (8). Nevertheless, the high proportion of multidrug-resistant backgrounds creates potential public health risks if further adaptive evolution emerges, reinforcing the necessity of continuous WGS-based epidemiological surveillance.

### Clinical and public health implications

Borderline *rpoB* variants with genotype–phenotype discordance deserve regular surveillance, and middle-aged diabetic males constitute a priority population for targeted screening and prevention.

Phenotypic resistance from these mutations often presents as low-level or borderline, easily overlooked by conventional solid-medium DST (18). Accordingly, BMD supplementary MIC detection is advised for isolates with Xpert-predicted resistance but susceptible phenotype, with WGS preferred to clarify mutation genotype and accompanying resistance mutations. MIC testing is indispensable for WHO-listed Interim variants to validate their phenotypic impact and helps stratify resistance severity for AwR-classified borderline substitutions (17). Consistent with prior research, sub-breakpoint elevated RIF/INH MIC independently raises treatment failure and relapse risk (28); hence, conventional binary susceptible/resistant reporting cannot reflect the hidden clinical risks of borderline resistance.

Given frequent co-resistance to multiple anti-tuberculosis drugs among borderline-mutant isolates, we propose a clear management pathway: immediate supplementary molecular or phenotypic DST for fluoroquinolone and INH resistance is required once any *rpoB* variant is detected and confirmed MDR/Pre-XDR-TB should be managed per WHO therapeutic specifications (29). For rare isolates solely harboring low-risk Interim mutations without additional resistance, personalized RIF dose adjustment demands rigorous risk–benefit evaluation. Given the inherent relapse risk of standard short-course chemotherapy (30, 31), off-standard intensified treatment is not recommended outside formal ethical clinical trials, as modified drug exposure may escalate selection pressure and drive further resistance evolution.

Despite the absence of recent transmission clusters supporting the fitness-cost hypothesis of borderline mutations (8), prevalent coexisting MDR/Pre-XDR background highlights potential public health hazards driven by subsequent adaptive evolution. We propose three-tiered comprehensive surveillance optimization: First, upgrade molecular diagnostic reporting: Xpert and sequencing-based tests should specify exact mutation sites rather than only binary resistance outcomes to facilitate precise clinical judgment. Second, optimize laboratory and population surveillance: incorporate MIC data of genotype–phenotype discordant isolates into routine resistance surveillance; deploy sustained WGS monitoring for genomic evolution; and launch multidisciplinary cohort studies to evaluate long-term epidemiological risks of borderline-resistant strains.

Collectively, these proposals build a full-chain management system covering diagnosis, clinical therapy and public health control to curb the further spread and evolution of resistant TB.

### Limitations and future perspectives

This work constitutes the first systematic molecular epidemiological investigation of borderline *rpoB*-mutant *M. tuberculosis* in Changchun and advances understanding of genotype–phenotype inconsistency, with several limitations guiding subsequent research directions.

First, the single-center retrospective design and limited sample size may affect generalizability; larger multi-center prospective studies are needed to confirm the epidemiological prevalence and mutation spectrum across Northeast China.

Second, unavailable clinical follow-up data prevents evaluation of mutation-associated treatment failure or relapse. Longitudinal studies linking genotype, phenotypic profile, and therapeutic outcomes are necessary to facilitate precision clinical management.

Third, while genomic associations identified key mutations, the exact molecular mechanisms underlying low-level resistance and fitness changes remain to be elucidated. Future studies should combine *in vitro* recombinant protein assays, molecular dynamics simulations, and quantitative bacterial phenotyping to clarify the biochemical and cellular basis of borderline mutations.

Lastly, despite absent recent transmission events in our cohort, the prevalent Pre-XDR/MDR background necessitates persistent monitoring. Prospective epidemiological tracking paired with ongoing WGS surveillance will clarify long-term evolutionary trends and public health risks of these mutants.

In conclusion, our study establishes baseline epidemiological evidence for borderline RIF resistance. Follow-up multidisciplinary research combining large-scale clinical enrollment, patient outcome monitoring, functional experiments, and population surveillance will help translate laboratory findings into refined TB prevention and control strategies.

## Supplementary Material

Reviewer comments

## Data Availability

All raw WGS data generated in this study have been deposited in the NCBI Sequence Read Archive (SRA) under BioProject accession number PRJNA1460540. The BioSample accession numbers, complete L-J DST results and BASO MIC values for all tested drugs of individual isolates are summarized in Table S1.

## SUPPLEMENTAL MATERIAL

Supplemental Material spectrum.00327-26-s0001.docx Tables S1 and S2.

OPEN PEER REVIEW reviewer-comments.pdf An accounting of the reviewer comments and feedback.

ASM does not own the copyrights to Supplemental Material that may be linked to, or accessed through, an article. The authors have granted ASM a non-exclusive, world-wide license to publish the Supplemental Material files. Please contact the corresponding author directly for reuse.
