# Supplementary material for: Genomic insights into low-level rifampicin resistance mediated by borderline rpoB mutations in Mycobacterium tuberculosis: prevalence and phylogeny in Northeast China
Source: Microbiol Spectr. 2026 Jul 21;14(9):e00327-26. doi: 10.1128/spectrum.00327-26 (PMC13532235; doi:10.1128/spectrum.00327-26)
Supplement: Supplementary file 1 [file reviewer-comments.pdf]

AMERICAN SOCIETY FOR MICROBIOLOGY | Microbiology Spectrum

Genomic Insights into Low-Level Rifampicin Resistance Mediated by Borderline rpoB Mutations in Mycobacterium tuberculosis: Prevalence and Phylogeny in Northeast China

Lingling Li, Ming-Jin Zhang, Lin Su, Xiaoting Qin, Huiying Liu, Xue Zhang, Kewei Liu, Xuejuan Sun, and Feng Yan

Corresponding Author(s): Lingling Li, Changchun Infectious Disease Hospital

Review Timeline:

Submission Date:	January 25, 2026
Editorial Decision:	April 24, 2026
Revision Received:	May 8, 2026
Editorial Decision:	May 24, 2026
Revision Received:	June 11, 2026
Editorial Decision:	June 12, 2026
Revision Received:	June 16, 2026
Accepted:	June 24, 2026

Editor: Ping Ren

Reviewer(s): The reviewers have opted to remain anonymous.

Transaction Report:

(Note: With the exception of the correction of typographical or spelling errors that could be a source of ambiguity, letters and reports are not edited. The original formatting of letters and referee reports may not be reflected in this compilation.)

DOI: https://doi.org/10.1128/spectrum.00327-26

Re: Spectrum00327-26 (Genomic Insights into Low-Level Rifampicin Resistance Mediated by Borderline rpoB Mutations in Mycobacterium tuberculosis: Prevalence and Phylogeny in Northeast China)

Dear Dr. Feng Yan:

Thank you for the privilege of reviewing your work. Below you will find my comments, instructions from the Spectrum editorial office, and the reviewer comments.

Please return the manuscript within 60 days; if you cannot complete the modification within this time period, please contact me. If you do not wish to modify the manuscript and prefer to submit it to another journal, notify me immediately so that the manuscript may be formally withdrawn from consideration by Spectrum.

Revision Guidelines
To submit your modified manuscript, log into the submission site at https://spectrum.msubmit.net/cgi-bin/main.plex. Go to Author Tasks and click the appropriate manuscript title to begin. The information you entered when you first submitted the paper will be displayed; update this as necessary. Note the following requirements:

• Upload point-by-point responses to the issues raised by the reviewers in a file named "Response to Reviewers," NOT in your cover letter.
• Upload a compare copy of the manuscript (without figures) as a "Marked-Up Manuscript" file.
• Upload a clean .DOC/.DOCX version of the revised manuscript and remove the previous version.
• Each figure must be uploaded as a separate, editable, high-resolution file (TIFF or EPS preferred), and any multipanel figures must be assembled into one file.
• Any supplemental material intended for posting by ASM should be uploaded with their legends separate from the main manuscript. You can combine all supplemental material into one file (preferred) or split it into a maximum of 10 files with all associated legends included.

For complete guidelines on revision requirements, see our Submission and Review Process webpage. Submission of a paper that does not conform to guidelines may delay acceptance of your manuscript.

Data availability: ASM policy requires that data be available to the public upon online posting of the article, so please verify all links to sequence records, if present, and make sure that each number retrieves the full record of the data. If a new accession number is not linked or a link is broken, provide Spectrum production staff with the correct URL for the record. If the accession numbers for new data are not publicly accessible before the expected online posting of the article, publication may be delayed; please contact production staff (Spectrum@asmusa.org) immediately with the expected release date.

Publication Fees: For information on publication fees and which article types are subject to charges, visit our website. If your manuscript is accepted for publication and any fees apply, you will be contacted separately about payment during the production process; please follow the instructions in that e-mail. Arrangements for payment must be made before your article is published.

ASM Membership: Corresponding authors may join or renew ASM membership to obtain discounts on publication fees. Need to upgrade your membership level? Please contact Customer Service at Service@asmusa.org.

The ASM Journals program strives for constant improvement in our submission and publication process. Please tell us how we can improve your experience by taking this quick Author Survey.

Thank you for submitting your paper to Spectrum.

Sincerely,
Ping Ren
Editor
Microbiology Spectrum

Reviewer #2 (Comments for the Author):

According to the data availability section, Li et al. will only share de-identified data provided that a data use agreement is signed. This is part of a wider pattern according to which researchers from Chinese institutions publish genomic studies for TB without depositing the underlying genomes. In my view, this is not acceptable, and I will not review this manuscript until a supplementary file has been prepared that relates all MICs and mutations discussed in the manuscript with the accession numbers of the

deposited genomes.

Response to Reviewer #2:

We sincerely appreciate the reviewer's critical comment regarding data availability. We have addressed this concern comprehensively through the following revisions:

1、Public deposition of whole-genome sequencing data

All raw whole-genome sequencing data for the isolates included in this study have been successfully deposited in the NCBI Sequence Read Archive (SRA) under the BioProject accession number PRJNA1460540 (released on 2026-05-02; available at https://www.ncbi.nlm.nih.gov/sra/PRJNA1460540). All data are publicly accessible without any restrictions or data use agreements.

2、Correlation of MIC, mutations, and accession numbers

We have added Table S1 to the supplementary materials, which clearly links each isolate ID, corresponding rpoB mutation, rifampicin MIC value, and BioSample accession number. This directly addresses the requirement to associate phenotypic and genotypic data with the deposited genomes.

3、Updated Data Availability statement in the manuscript

A formal Data Availability section has been added at the end of the MATERIALS AND METHODS section, providing full details of the data deposition, accession number, and public release status.

We hope these revisions fully address the reviewer’s concerns.

Thank you again for your time and consideration.

Sincerely,

Lingling Li

Re: Spectrum00327-26R1 (Genomic Insights into Low-Level Rifampicin Resistance Mediated by Borderline rpoB Mutations in Mycobacterium tuberculosis: Prevalence and Phylogeny in Northeast China)

Dear Dr. Lingling Li:

Thank you for the privilege of reviewing your work. Below you will find my comments, instructions from the Spectrum editorial office, and the reviewer comments.

Please return the manuscript within 60 days; if you cannot complete the modification within this time period, please contact me. If you do not wish to modify the manuscript and prefer to submit it to another journal, notify me immediately so that the manuscript may be formally withdrawn from consideration by Spectrum.

Revision Guidelines
To submit your modified manuscript, log into the submission site at https://spectrum.msubmit.net/cgi-bin/main.plex. Go to Author Tasks and click the appropriate manuscript title to begin. The information you entered when you first submitted the paper will be displayed; update this as necessary. Note the following requirements:

• Upload point-by-point responses to the issues raised by the reviewers in a file named "Response to Reviewers," NOT in your cover letter.
• Upload a compare copy of the manuscript (without figures) as a "Marked-Up Manuscript" file.
• Upload a clean .DOC/.DOCX version of the revised manuscript and remove the previous version.
• Each figure must be uploaded as a separate, editable, high-resolution file (TIFF or EPS preferred), and any multipanel figures must be assembled into one file.
• Any supplemental material intended for posting by ASM should be uploaded with their legends separate from the main manuscript. You can combine all supplemental material into one file (preferred) or split it into a maximum of 10 files with all associated legends included.

For complete guidelines on revision requirements, see our Submission and Review Process webpage. Submission of a paper that does not conform to guidelines may delay acceptance of your manuscript.

Data availability: ASM policy requires that data be available to the public upon online posting of the article, so please verify all links to sequence records, if present, and make sure that each number retrieves the full record of the data. If a new accession number is not linked or a link is broken, provide Spectrum production staff with the correct URL for the record. If the accession numbers for new data are not publicly accessible before the expected online posting of the article, publication may be delayed; please contact production staff (Spectrum@asmusa.org) immediately with the expected release date.

Publication Fees: For information on publication fees and which article types are subject to charges, visit our website. If your manuscript is accepted for publication and any fees apply, you will be contacted separately about payment during the production process; please follow the instructions in that e-mail. Arrangements for payment must be made before your article is published.

ASM Membership: Corresponding authors may join or renew ASM membership to obtain discounts on publication fees. Need to upgrade your membership level? Please contact Customer Service at Service@asmusa.org.

The ASM Journals program strives for constant improvement in our submission and publication process. Please tell us how we can improve your experience by taking this quick Author Survey.

Thank you for submitting your paper to Spectrum.

Sincerely,
Ping Ren
Editor
Microbiology Spectrum

Reviewer #2 (Comments for the Author):

The study by Li et al. features interesting results relating to discordant rifampicin results but needs to be improved.

Major comments:
1. Most international readers are likely not familiar with the Beisuo BMD plates. Therefore, please explain:

a. The concentration range for RIF on the plate;
b. The regulatory status in China of the plate;
c. Why 1 mg/L was used for the interpretation.
2. Was H37Rv included in every batch of BMD testing? What MICs were obtained?
3. Please explain what you mean by "heteroresistance-related" false positives.
4. Update Fig. 1 to include genotypic predictions for all currently recommended drugs and remove those that are not (i.e. capreomycin). For example, it is not clear to me whether the two Leu452Met mutants are also genotypically susceptible to pyrazinamide.
5. Please state explicitly which mutations are officially recognized as borderline by WHO and that a composite reference standard has been endorsed (PMID 33568463).

Minor comments:
1. Species and gene names throughout the paper and references should be italicized (e.g. line 12 "Mycobacterium tuberculosis").
2. Spaces are missing through the manuscript (e.g. line 58 "Löwenstein-Jensen(L-J)").
3. There are several problems with the references (e.g. reference 34 in line 71 "included in the regimen 34" is missing as is reference 89 in line 81 "as mono-resistance 89").
4. What do you mean by "functional nonsynonymous mutations"?
5. Explain what the numbers on the branches of Fig. 1A represent.
6. The discussion repeats a lot of the results unnecessarily and needs to be shortened.

Major comments:

1. Most international readers are likely not familiar with the Beisuo BMD plates. Therefore, please explain:

For clarity, “Beisuo” is the Chinese phonetic spelling of “BASO”, the official brand name of the commercial BMD plates used in this study. We have revised the manuscript to use BASO consistently throughout the text.

a. The concentration range for RIF on the plate.

According to the manufacturer’s specifications, the rifampicin (RIF) concentrations on the BASO BMD plate are 0.25, 0.5, 1, 2, 4, and 8 mg/L.

This content has been added on Page 8, Lines 155–156 in the MATERIALS AND METHODS section of the manuscript.

b. The regulatory status in China of the plate.

The BMD plates (BASO Diagnostics, Zhuhai, China) are NMPA-registered Class II in vitro diagnostic devices in China, with the registration number Yue Medical Device Registration Certificate No. 20182400952. They are commercially available and widely used in routine clinical laboratories across China for Mycobacterium tuberculosis susceptibility testing, complying with national regulatory and laboratory standards.

c. Why 1 mg/L was used for the interpretation

We used 1 mg/L as the interpretive breakpoint in accordance

with the requirements of the BASO BMD kit certified by the National Medical Products Administration. Although the WHO-recommended rifampicin breakpoint of 0.5 mg/L has passed manufacturer validation (including registration tests and clinical trials) and completed formal submission, this standard is still pending final approval by domestic regulatory authorities.

The above content has been added on Page 8, the section of MIC Determination in MATERIALS AND METHODS (Lines 156–161).

2. Was H37Rv included in every batch of BMD testing? What MICs were obtained?

H37Rv was included as a quality control strain in all three batches of BMD testing. The RIF MIC values for H37Rv were consistently <0.25 mg/L across all batches, confirming reliable and reproducible test performance.

3. Please explain what you mean by "heteroresistance-related" false positives.

These false positives refer to inconsistent rifampicin resistance results between the initial Xpert MTB/RIF test on clinical specimens and repeat testing using pure cultures. To verify the reliability of genotypic resistance results, all candidate isolates were validated: first, the rifampicin MIC of all isolates was determined to be ≤1 μg/mL by BMD; second, repeat Xpert MTB/RIF testing on pure

cultures failed to detect rifampicin resistance in three isolates, which was inconsistent with the initial results. All three isolates presented five positive probes with ΔCt ($Ct_{max} - Ct_{min}$) > 4, indicating potential heteroresistance (coexistence of susceptible and resistant subpopulations). As suggested by Ocheretina et al., low bacterial load in clinical specimens may capture low-abundance non-functional mutant subclones, which are often outcompeted by susceptible clones during subsequent pure culture, leading to unconfirmed resistance upon retesting. To ensure accurate genotypic data, these three isolates were excluded.

The above revisions have been made on Pages 6–7, in the Study Isolates subsection of the MATERIALS AND METHODS section (Lines 122–135).

4. Update Fig. 1 to include genotypic predictions for all currently recommended drugs and remove those that are not (i.e. capreomycin). For example, it is not clear to me whether the two Leu452Met mutants are also genotypically susceptible to pyrazinamide.

Figure 1 has been revised accordingly: genotypic profiles of recommended anti-TB agents (including PZA, BDQ, LZD, DLM and CFZ) were supplemented, and capreomycin was removed from the dataset. Of note, phenotypic drug susceptibility testing for BDQ, LZD, DLM and CFZ was not performed in this study because these four novel anti-tuberculosis drugs have not been approved for

routine clinical susceptibility testing.In addition, all five isolates carrying the Leu452Met mutation were phenotypically and genotypically susceptible to pyrazinamide, which has been updated in the revised fig 1.

5. Please state explicitly which mutations are officially recognized as borderline by WHO and that a composite reference standard has been endorsed (PMID 33568463).

We have specified the WHO-recognized borderline mutations and the endorsed composite reference standard in the Discussion: According to Köser et al. (2021, PMID 33568463), seven borderline rpoB mutations (Leu430Pro, Asp435Tyr, His445Leu, His445Asn, His445Ser, Leu452Pro, Ile491F) are officially recognized by WHO as clinically relevant to rifampicin resistance. In line with the WHO-endorsed composite reference standard (expert rule), an isolate is defined as RIF-resistant if it shows phenotypic resistance, carries any of the seven borderline mutations, or bears any nonsynonymous mutation within the rpoB RRDR region.

The relevant content has been revised on Page 13, in the subsection Prevalence of Borderline rpoB Mutations and Limitations of Phenotypic Testing within the DISCUSSION and MATERIALS AND METHODS sections (Lines 275–280).

Minor comments:

1. Species and gene names throughout the paper and references should be italicized (e.g. line 12 "Mycobacterium tuberculosis").

All species and gene names, including Mycobacterium tuberculosis and gene symbols such as rpoB and gyrA, as well as amino acid variants (e.g., Leu452Pro, Leu430Pro), have been properly italicized in the revised manuscript.

2. Spaces are missing through the manuscript (e.g. line 58 "Löwenstein-Jensen(L-J)").

Missing spaces throughout the manuscript (such as "Löwenstein-Jensen(L-J)" on line 58) have been fully checked and corrected.

3. There are several problems with the references (e.g. reference 34 in line 71 "included in the regimen 34" is missing as is reference 89 in line 81 "as mono-resistance 89").

All reference citation errors (missing citations for Ref.34 at line 71 and Ref.89 at line 81) have been checked and amended throughout the manuscript.

4. What do you mean by "functional nonsynonymous mutations"?

We have added a definition of functional nonsynonymous mutations in the revised manuscript where the term first appears. This term describes nonsynonymous mutations that alter amino acid sequences and carry proven or potential functional consequences such as antimicrobial resistance.

In our study, besides resistance-conferring mutations for other anti-TB drugs (e.g., gyrA Asp94Ala, katG Ser315Asn, gyrA Asp94Gly), six distinct rpoB variants related to rifampicin were identified: Leu452Pro, Leu430Pro, His445Asn, Leu452Met, Asp435Ala and Asp435Gly. According to the WHO mutation catalogue, Leu452Pro, Leu430Pro and His445Asn are graded as "Associated with resistance (AwR)", whereas Leu452Met, Asp435Ala and Asp435Gly belong to the "Assoc w R - Interim" group. Per WHO's classification standard, all these variants are defined as functional nonsynonymous mutations due to their annotated definite or potential ability to induce rifampicin resistance.

The corresponding explanation has been added on Page 10, Lines 208–210 of the Genotypic Characteristics subsection in the RESULTS section.

5. Explain what the numbers on the branches of Fig. 1A represent.

We have updated the legend of Figure 1A to specify the branch numbers: Values shown along tree branches denote branch lengths, which represent genetic distance calculated from core-genome SNPs, reflecting core-genome single-nucleotide polymorphism divergence between clades.

The corresponding explanation has been added to the notes of Figure 1.

6. The discussion repeats a lot of the results unnecessarily and needs to be shortened.

We have carefully revised and condensed the Discussion section to remove redundant content that overlaps with the Results, and streamlined the whole section appropriately.

Re: Spectrum00327-26R2 (Genomic Insights into Low-Level Rifampicin Resistance Mediated by Borderline rpoB Mutations in Mycobacterium tuberculosis: Prevalence and Phylogeny in Northeast China)

Dear Dr. Lingling Li:

Thank you for the privilege of reviewing your work. Below you will find my comments, instructions from the Spectrum editorial office, and the reviewer comments.

Please return the manuscript within 60 days; if you cannot complete the modification within this time period, please contact me. If you do not wish to modify the manuscript and prefer to submit it to another journal, notify me immediately so that the manuscript may be formally withdrawn from consideration by Spectrum.

Revision Guidelines
To submit your modified manuscript, log into the submission site at https://spectrum.msubmit.net/cgi-bin/main.plex. Go to Author Tasks and click the appropriate manuscript title to begin. The information you entered when you first submitted the paper will be displayed; update this as necessary. Note the following requirements:

• Upload point-by-point responses to the issues raised by the reviewers in a file named "Response to Reviewers," NOT in your cover letter.
• Upload a compare copy of the manuscript (without figures) as a "Marked-Up Manuscript" file.
• Upload a clean .DOC/.DOCX version of the revised manuscript and remove the previous version.
• Each figure must be uploaded as a separate, editable, high-resolution file (TIFF or EPS preferred), and any multipanel figures must be assembled into one file.
• Any supplemental material intended for posting by ASM should be uploaded with their legends separate from the main manuscript. You can combine all supplemental material into one file (preferred) or split it into a maximum of 10 files with all associated legends included.

For complete guidelines on revision requirements, see our Submission and Review Process webpage. Submission of a paper that does not conform to guidelines may delay acceptance of your manuscript.

Data availability: ASM policy requires that data be available to the public upon online posting of the article, so please verify all links to sequence records, if present, and make sure that each number retrieves the full record of the data. If a new accession number is not linked or a link is broken, provide Spectrum production staff with the correct URL for the record. If the accession numbers for new data are not publicly accessible before the expected online posting of the article, publication may be delayed; please contact production staff (Spectrum@asmusa.org) immediately with the expected release date.

Publication Fees: For information on publication fees and which article types are subject to charges, visit our website. If your manuscript is accepted for publication and any fees apply, you will be contacted separately about payment during the production process; please follow the instructions in that e-mail. Arrangements for payment must be made before your article is published.

ASM Membership: Corresponding authors may join or renew ASM membership to obtain discounts on publication fees. Need to upgrade your membership level? Please contact Customer Service at Service@asmusa.org.

The ASM Journals program strives for constant improvement in our submission and publication process. Please tell us how we can improve your experience by taking this quick Author Survey.

Thank you for submitting your paper to Spectrum.

Sincerely,
Ping Ren
Editor
Microbiology Spectrum

Reviewer #2 (Comments for the Author):

Major comments:
1. You had three isolates that were initially Xpert-resistant, which could not be confirmed upon repeat testing. Your conclusion that these were "heteroresistance-resistance false positives" is not justified as you did not carry out sequencing to check whether a mutation was present or not. In fact, if a resistance mutation had been present even below 100% (e.g. at an allele

frequency of 50%), this would have been a true-positive result. What were the initial bacillary load results? If they were low or very low, the most likely explanation for the results is that they were cutoff errors because the bacillary load was close to the LoD of the assay (PMID 34686228 and 34615575). Otherwise, these were likely random false-resistant results.
2. Add the crucial concentrations for all drugs tested with LJ and update Table S1 to include all LJ DST results and the BASO MICs for all drugs. Also, add a supplementary table that shows all drugs and concentrations included on the plate and the breakpoint for interpretation.

Minor comments:
1. Please include the H37Rv MICs for RIF in the manuscript as well rather just in response to my last review.
2. Given the small sample size, please round percentages to the nearest integer.

Major comments:

1. You had three isolates that were initially Xpert-resistant, which could not be confirmed upon repeat testing. Your conclusion that these were "heteroresistance-resistance false positives" is not justified as you did not carry out sequencing to check whether a mutation was present or not. In fact, if a resistance mutation had been present even below 100% (e.g. at an allele frequency of 50%), this would have been a true-positive result. What were the initial bacillary load results? If they were low or very low, the most likely explanation for the results is that they were cutoff errors because the bacillary load was close to the LoD of the assay (PMID 34686228 and 34615575). Otherwise, these were likely random false-resistant results.

We appreciate the comment and have updated the manuscript as suggested.

Detailed descriptions of the three discrepant isolates, including their initial bacillary loads (one "low" and two "very low") and the underlying mechanism of test discrepancy, have been inserted into Materials and Methods, Study Isolates (Page 6, Lines 126–131). The conflicting Xpert results are explained by cut-off errors: bacillary loads near the assay LoD cause unstable amplification and hybridization signals, resulting in inconsistent readings, poor reproducibility and false resistance calls (PMID: 34686228, 34615575).

2. Add the crucial concentrations for all drugs tested with LJ and update Table S1 to include all LJ DST results and the BASO MICs for all drugs. Also, add a supplementary table that shows all drugs and concentrations included on the plate and the breakpoint for interpretation.

1) We have added the critical concentrations of all tested drugs for the L-J proportion method in the Bacterial Culture and Identification

subsection, Laboratory Methods (Page 7, Lines 146–150).

2) Table S1 has been revised to list all drug susceptibility results obtained via the L-J proportion method and MIC values detected using BASO reagents. The relevant content has been cited in the MIC Determination subsection, Laboratory Methods (Page 8, Lines 166–167).

3) A new supplementary table (Table S2) has been created to list all tested anti-tuberculosis drugs, their serial concentrations on the commercial assay plates, and the corresponding interpretive breakpoints. Furthermore, we have described the discrepancies between the breakpoints of the commercial kits and the WHO-recommended epidemiological cut-off values, along with the potential impacts of these differences on our experiments. All related revisions have been made in the MIC Determination subsection, Laboratory Methods (Page 8, Lines 156–168).

Minor comments:

1. Please include the H37Rv MICs for RIF in the manuscript as well rather just in response to my last review.

We have added the RIF MIC data of the quality control strain Mycobacterium tuberculosis H37Rv in the MIC Determination subsection, Laboratory Methods (Page 8, Lines 174–176).

2. Given the small sample size, please round percentages to the nearest integer.

We have rounded all percentage values in the manuscript to the nearest integer, and added a relevant explanation in the Descriptive Statistics subsection, Laboratory Methods (Page 9, Lines 193–194).

Re: Spectrum00327-26R3 (Genomic Insights into Low-Level Rifampicin Resistance Mediated by Borderline rpoB Mutations in Mycobacterium tuberculosis: Prevalence and Phylogeny in Northeast China)

Dear Dr. Lingling Li:

Your manuscript has been accepted, and I am forwarding it to the ASM production staff for publication. Your paper will first be checked to make sure all elements meet the technical requirements. ASM staff will contact you if anything needs to be revised before copyediting and production can begin. Otherwise, you will be notified when your proofs are ready to be viewed.

Data Availability: ASM policy requires that data be available to the public upon online posting of the article, so please verify all links to sequence records, if present, and make sure that each number retrieves the full record of the data. If a new accession number is not linked or a link is broken, provide production staff with the correct URL for the record. If the accession numbers for new data are not publicly accessible before the expected online posting of the article, publication may be delayed; please contact ASM production staff immediately with the expected release date.

Publication Fees: For information on publication fees and which article types have charges, please visit our website. We have partnered with Copyright Clearance Center (CCC) to collect author charges. If fees apply to your paper, you will receive a message from no-reply@copyright.com with further instructions. For questions related to paying charges through RightsLink, please contact CCC at ASM_Support@copyright.com or toll free at +1-877-622-5543. CCC makes every attempt to respond to all emails within 24 hours.

ASM Membership: Corresponding authors may join or renew ASM membership to obtain discounts on publication fees. Need to upgrade your membership level? Please contact Customer Service at Service@asmusa.org.

Embargo Policy: A press release may be issued as soon as the manuscript is posted on the Spectrum Latest Articles webpage. The corresponding author will receive an email with the subject line "ASM Journals Author Services Notification" when the article is available online.

The ASM Journals program strives for constant improvement in our submission and publication process. Please tell us how we can improve your experience by taking this quick Author Survey.

Thank you for submitting your paper to Spectrum.

Sincerely,
Ping Ren
Editor
Microbiology Spectrum
