# Supplementary material for: Genomic insights into low-level rifampicin resistance mediated by borderline rpoB mutations in Mycobacterium tuberculosis: prevalence and phylogeny in Northeast China
Source: Microbiol Spectr. 2026 Jul 21;14(9):e00327-26. doi: 10.1128/spectrum.00327-26 (PMC13532235; doi:10.1128/spectrum.00327-26)
Supplement: Supplementary file 2 [file spectrum.00327-26-s0001.docx]

**Table S1. Genotypes, phenotypic drug susceptibility, and genome accession numbers of *Mycobacterium tuberculosis* isolates**

| **Sample ID** | ***rpoB* mutation** | **Other mutations** | **L-J DST (S/R) / MIC(mg/L)** | | | | | | | | | | **BioSample Accession** |
| --- | --- | --- | --- | --- | --- | --- | --- | --- | --- | --- | --- | --- | --- |
|  |  |  | **RIF** | **INH** | **EMB** | **PZA** | **LFX** | **MFX** | **AMK** | **PTO** | **SM** | **PAS** |  |
| **2312** | ***Leu452Pro*** | **ND** | **S/1** | **S/≤0.025** | **S/0.62** | **-/≤50** | **S/≤0.5** | **S/0.25** | **S/≤0.25** | **S/≤0.62** | **S/≤0.25** | **S/≤0.5** | **SAMN57600215** |
| **437** | ***Leu452Pro*** | **ND** | **S/1** | **S/≤0.025** | **S/0.62** | **-/≤50** | **S/≤0.5** | **S/0.25** | **S/≤0.25** | **S/≤0.62** | **S/≤0.25** | **S/2** | **SAMN57600214** |
| **072105** | ***Leu452Pro*** | ***gyrA Asp94Ala*** | **S/1** | **S/≤0.025** | **S/≤0.31** | **-/≤50** | **R/4** | **S/0.5** | **S/≤0.25** | **S/≤0.62** | **S/≤0.25** | **S/≤0.5** | **SAMN57600222** |
| **7288** | ***Leu452Pro*** | ***gyrA Asp94Ala*** | **S/1** | **S/≤0.025** | **S/0.62** | **-/≤50** | **R/4** | **R/2** | **S/≤0.25** | **S/≤0.62** | **S/≤0.25** | **S/2** | **SAMN57600223** |
| **13106** | ***Leu452Pro*** | ***katG Ser315Asn;***  ***gyrA Asp94Gly*** | **S/1** | **R/1** | **S/0.62** | **-/≤50** | **R/8** | **R/1** | **S/≤0.25** | **S/≤0.62** | **S/≤0.25** | **S/2** | **SAMN57600228** |
| **3360** | ***Leu430Pro*** | ***embB Met306Ile*** | **S/≤0.25** | **S/≤0.025** | **R/10** | **-/≤50** | **S/≤0.5** | **S/0.25** | **S/≤0.25** | **S/≤0.62** | **S/≤0.25** | **S/≤0.5** | **SAMN57600217** |
| **4129** | ***Leu430Pro*** | ***inhA c.-777C>T;***  ***gyrA Asp94Ala*;**  ***rpsL Lys43Arg;*** | **S/0.5** | **R/1** | **S/0.62** | **-/≤50** | **R/4** | **R/2** | **S/≤0.25** | **R/10** | **R/>40** | **R/>16** | **SAMN57600219** |
| **6056** | ***Leu430Pro*** | ***gyrA Asp94Asn*** | **S/0.5** | **S/≤0.025** | **S/0.62** | **-/≤50** | **R/8** | **R/4** | **S/≤0.25** | **S/≤0.62** | **S/≤0.25** | **S/2** | **SAMN57600224** |
| **6068** | ***Leu430Pro*** | ***katG Ser315Thr*;**  ***pncA Thr47Ala*;**  ***gyrA Asp94Asn*;**  ***rpsL Lys43Arg*** | **S/0.5** | **R/2** | **S/1.25** | **-/900** | **R/8** | **R/4** | **S/≤0.25** | **S/≤0.62** | **R/>40** | **S/2** | **SAMN57600225** |
| **14216** | ***Leu430Pro*** | ***gyrA Asp94Gly*** | **S/≤0.25** | **S/≤0.025** | **S/0.62** | **-/≤50** | **R/8** | **R/4** | **S/≤0.25** | **S/≤0.62** | **S/≤0.25** | **S/1** | **SAMN57600230** |
| **12821** | ***His445Asn*** | ***rpsL Lys43Arg*** | **S/≤0.25** | **S/≤0.025** | **S/0.62** | **-/≤50** | **S/1** | **S/0.25** | **S/≤0.25** | **S/≤0.62** | **R/>40** | **S/≤0.5** | **SAMN57600227** |
| **13693** | ***His445Asn*** | ***rpsL Lys43Arg*** | **S/≤0.25** | **S/≤0.025** | **S/0.62** | **-/≤50** | **S/≤0.5** | **S/0.25** | **S/≤0.25** | **S/≤0.62** | **R/>40** | **S/2** | **SAMN57600229** |
| **4058** | ***His445Asn*** | ***katG Ser315Thr*; *gidB c.102delG*** | **S/≤0.25** | **R/2** | **S/0.62** | **-/≤50** | **S/≤0.5** | **S/0.25** | **S/≤0.25** | **S/≤0.62** | **R/4** | **S/1** | **SAMN57600218** |
| **3120** | ***Leu452Met*** | **ND** | **S/≤0.25** | **S/≤0.025** | **S/0.62** | **-/≤50** | **S/≤0.5** | **S/0.25** | **S/≤0.25** | **S/≤0.62** | **S/≤0.25** | **S/2** | **SAMN57600216** |
| **4352** | ***Leu452Met*** | **ND** | **S/0.5** | **S/≤0.025** | **S/0.62** | **-/≤50** | **S/≤0.5** | **S/0.25** | **S/≤0.25** | **S/1.25** | **S/≤0.25** | **S/2** | **SAMN57600221** |
| **4346** | ***Asp435Ala*** | ***gyrA Asp94Gly*** | **S/≤0.25** | **S/0.05** | **S/0.62** | **-/≤50** | **R/8** | **R/2** | **S/≤0.25** | **S/≤0.62** | **S/≤0.25** | **S/1** | **SAMN57600220** |
| **10907** | ***Asp435Gly*** | ***gyrA Asp94Gly*** | **S/≤0.25** | **S/≤0.025** | **S/0.62** | **-/≤50** | **R/8** | **R/2** | **S/≤0.25** | **S/≤0.62** | **S/≤0.25** | **S/≤0.5** | **SAMN57600226** |

**Note: RIF, rifampicin; INH, isoniazid; EMB, ethambutol; PZA, pyrazinamide; LFX, levofloxacin; MFX, moxifloxacin; AMK, amikacin; PTO, prothionamide; SM, streptomycin; PAS, para-aminosalicylic acid. L-J DST results: S, susceptible; R, resistant. Symbol "−" indicates L-J DST was not performed for PZA; ND, not detected.**

**Table S2. Tested concentrations and interpretive breakpoints of anti-tuberculosis drugs on the BASO BMD plate**

| **Drug Abbreviation** | **Full Drug Name** | **Tested Concentrations (mg/L)** | **Interpretive Breakpoint (mg/L)** |
| --- | --- | --- | --- |
| INH | Isoniazid | 0.025, 0.05, 0.1, 0.2, 0.5, 1, 2, 4 | 0.2 |
| RIF | Rifampicin | 0.25, 0.5, 1, 2, 4, 8 | 1 |
| PZA | Pyrazinamide | 50, 100, 150, 300, 600, 900 | 100 |
| EMB | Ethambutol | 0.31, 0.62, 1.25, 2.5, 5, 10, 20 | 5 |
| LFX | Levofloxacin | 0.5, 1, 2, 4, 8, 16 | 2 |
| MFX | Moxifloxacin | 0.06, 0.12, 0.25, 0.5, 1, 2, 4 | 0.5 |
| AMK | Amikacin | 0.25, 0.5, 1, 2, 4, 8, 16 | 1 |
| PTO | Prothionamide | 0.62, 1.25, 2.5, 5, 10, 20 | 2.5 |
| PAS | Para-aminosalicylic acid | 0.5, 1, 2, 4, 8, 16 | 2 |
| SM | Streptomycin | 0.25, 0.5, 1, 2, 4, 10, 20, 40 | 2 |
| CPM | Capreomycin | 0.5, 1, 2, 4, 8, 16 | 2 |
| RBU | Rifabutin | 0.12, 0.25, 0.5, 1, 2, 4 | 0.5 |
| OFX | Ofloxacin | 0.25, 0.5, 1, 2, 4, 8, 16 | 2 |
| KAN | Kanamycin | 0.62, 1.25, 2.5, 5, 10, 20 | 5 |

**Note: All drugs shown in this table are pre-coated on the commercial BASO BMD plate. Ten drugs were included for analysis in this study, while OFX, KAN, CPM and RBU were not analyzed.**
